# Comparative Transcriptome Analysis of the Cosmopolitan Marine Fungus *Corollospora maritima* Under Two Physiological Conditions

**DOI:** 10.1534/g3.115.019620

**Published:** 2015-06-26

**Authors:** Patricia Velez, Naholi D. Alejandri-Ramírez, María C. González, Karel J. Estrada, Alejandro Sanchez-Flores, Tzvetanka D. Dinkova

**Affiliations:** * Departamento de Botánica, Instituto de Biología, Universidad Nacional Autónoma de México, Distrito Federal, México 04510; † Departamento de Bioquímica, Facultad de Química, Universidad Nacional Autónoma de México, Distrito Federal, México 04510; ‡ Unidad de Secuenciación Masiva y Bioinformática Instituto de Biotecnología, Universidad Nacional Autónoma de México, Cuernavaca, Morelos, México 62210

**Keywords:** environmental stress, fungal transcriptomics, *Halosphaeriaceae*, intertidal zone, sandy seashore

## Abstract

Marine sandy beaches represent dynamic environments often subject to harsh conditions and climate fluctuations, where natural and anthropogenic inputs of freshwater from fluvial and pluvial sources alter salinity, which has been recognized as a key variable affecting the distribution of aquatic organisms and influencing critical physiological processes. The marine arenicolous fungus *Corollospora maritima* is a worldwide-distributed saprobe that has been reported to present tolerance to freshwater. Here, we present a transcriptome analysis that will provide the first insight of the genomic content for this fungus and a gene expression comparison between two different salinity conditions. We also identified genes that are candidates for being differentially expressed in response to environmental variations on salinity during the fungal growth. The *de novo* reconstruction of *C. maritima* transcriptome Illumina sequencing provided a total of 14,530 transcripts (16 megabases). The comparison between the two growth conditions rendered 103 genes specifically overexpressed in seawater, and 132 genes specifically up-regulated under freshwater. Using fungal isolates collected from different beaches, the specific environmental regulation of particular transcript differential expression was confirmed by RT-qPCR. To our knowledge, this is the first analysis that explores the marine fungus *C. maritima* molecular responses to overcome freshwater stress, and these data could shed light to understand the fungal adaptation and plasticity mechanisms to the marine habitat.

Sandy beaches represent the largest coastal environment on earth, covering 70% of all continental margins. This ecosystem performs critical habitat functions and links marine and terrestrial food webs (McLachlan and Brown 2006; Schlacher and Connolly 2009). Moreover, these beaches represent dynamic environments often subject to harsh conditions and climate fluctuations. Natural inputs of freshwater are common, originated from fluvial discharges and from pluvial source during rainy season. This input of freshwater to the beach alters salinity, which has been identified as a key variable affecting the distribution of aquatic organisms and influencing critical physiological processes (Day *et al.* 1989; Defeo and McLachlan 2005). Furthermore, some surveys have revealed that variations in salinity resulting from local freshwater discharges produce dramatic effects in the habitat and the resident macrofauna (Lercari and Defeo 2006).

The intertidal biodiversity provides marine beaches with ecological services not available through other ecosystems (McLachlan and Brown 2006). Marine arenicolous fungi are a key component of coastal biodiversity. This ecological group of saprobiotic fungi lives interstitially between or on the surface of sand grains, promoting organic degradation and mineralization of substrata containing cellulose, hemicelluloses, and lignin; they also represent important dietary elements for a variety of marine organisms (Kohlmeyer and Kohlmeyer 1979; Hyde *et al.* 1998; González and Hanlin 2010). These fungi are entirely adapted to inhabit the dynamic ecosystem that sandy beaches provide, because their morphology and life cycle are adjusted to the characteristics of this ecotone. However, knowledge about their physiological adaptations to marine intertidal environment is scarce.

Empirical information has showed that salinity is a central factor determining the geographical distribution of marine fungi and plays an important role in the morphology of some species (Hughes 1986; Nakagiri and Ito 1994; Jones 2000). However, other reports indicate that marine ascomycetes have a wide tolerance to low-salinity conditions (Byrne and Jones 1975). Decisively, there is no conclusive experimental information about the role that variations in salinity play in the physiology of marine fungi.

The marine arenicolous cosmopolitan species *Corollospora maritima* Werderm represents a useful model to analyze the effects that fluctuations in salinity have on the physiology of marine fungi. This dominant species has a worldwide geographical distribution and has been reported to be a freshwater-tolerant species (Jones and Jennings 1965). Additionally, it is useful for measuring changes and evaluating the ecological disturbance on coastal sandy beaches due to its moderate sensitivity to pollution. It has also been recognized to produce a new phthalide exhibiting antibacterial activity against *Staphylococcus aureus* and other microorganisms, and it represents a hydrocarbonoclastic species able to use n-hexadecane, n-tetradecane, L-hexadecene, and pristane as sole carbon sources for growth (Kohlmeyer and Kohlmeyer 1979; Kirk *et al.* 1991; Liberra *et al.* 1998; González and Hanlin 2010).

Molecular studies with *C. maritima* have mostly focused on the taxonomy of this ascomycete (Jones 2000; Kohlmeyer *et al.* 2000), reporting it as a member of the family *Halosphaeriaceae*, within the class *Sordariomycetes* (Jones *et al.* 2009). No data regarding key genes involved in the fungus adaptation on salinity fluctuations have been reported. Furthermore, the genome sequence of this species is not available. However, through a global analysis of gene expression at the RNA level, the understanding of gene function is possible. With the achievement of the sequencing of approximately 497 fungal genomes (http://www.ncbi.nlm.nih.gov) and the ongoing 1000 Fungal Genomes project by the JGI (Grigoriev *et al.* 2014), the examination of global changes in gene expression is an advantageous method for dissecting the molecular basis of adaptation. The emerging field of fungal transcriptomics has made significant progress in investigating economically important fungal pathogens, evaluating the fungal response to antifungal compounds, and analyzing lignocellulose-degrading mechanisms (Bhadauria *et al.* 2007; Martinez *et al.* 2009; Gao *et al.* 2011; Cornman *et al.* 2012). Therefore, the application of this approach becomes very useful to understand the biochemical mechanisms underlying the adaptation of some fungal species to marine habitats.

Here, we performed a transcriptome analysis of *C. maritima* grown under two different salinity conditions to explore the genetic basis of the adaptation of this fungus to marine habitats. By assessing the differential gene expression depending on salinity, we approached the molecular basis of *C. maritima* tolerance to salinity fluctuations. Selected individual genes preferentially expressed under freshwater or seawater growth were confirmed in independent biological replicates, as well as using geographically different isolates of the fungus. This study represents the first transcriptome analysis of *C. maritima* and explores the differential gene expression in response to salinity as a parameter of fungal adaptation to its environment.

## Materials and Methods

### Sampling

The following sandy beaches located in the Gulf of Mexico were sampled according to the procedures described by Kohlmeyer and Kohlmeyer (1979) during low tide: Pico de Oro (18° 27′ 0.6″ N, 92° 52′ 14.8″ W); Paraíso (18° 26′ 19″ N, 93° 13′ 4.3″ W); and Boca del Río (19° 07′ 19.97″ N, 96° 06′ 17.11″ W). A sample of washed-up detritus (consisting of driftwood, decayed sea grasses, and algae) was collected randomly in the intertidal zone of each one of the beaches and placed in Ziploc plastic bags. In the laboratory, the collected samples were incubated for 12 months and examined monthly for the presence of reproductive structures. To identify the recovered fungi, ascomata, asci, and ascospores were examined and measured using a Nikon Eclipse 80i. The publications of Kohlmeyer and Kohlmeyer (1979), Kohlmeyer and Volkmann-Kohlmeyer (1991), and Jones *et al.* (2009) were used to identify the fungi.

### Growth curves comparison

Prior to RNA isolation, we tested the growth rate of *C. maritima* strains isolated from Paraíso Beach in the Gulf of Mexico using three replicates per condition (marine and freshwater) to test the optimal RNA collection point. Growth rate was measured daily by registering the colony diameter on agar potato-dextrose medium under two salinity conditions (marine and freshwater) at 25°. For the marine condition we added artificial seawater (Instant Ocean; Aquarium Systems, USA) to the medium following the instructions given by the manufacturer. Statistical significance, in terms of differences in the rate of growth between the two conditions, was assessed using the "compareGrowthCurves" function of the StatMod software package for R (http://bioinf.wehi.edu.au/software/compareCurves) (Smyth *et al.* 2011). A statistical permutation test was performed to compare *C. maritima* colonies over the course of growth. The test statistic (mean *t*) was calculated as the mean of the pooled two-sample *t*-statistic between the colony diameters at each time weighted by the degrees of freedom. A *P*-value was obtained for the test statistic by simulation. Colonies were randomly allocated to each of the two groups and the mean *t* was recalculated for 10,000 data sets permuted in this way. The *P*-value is the proportion of permutations, where the mean *t* is greater in absolute value than the mean *t* for the original data set. Pairwise comparisons were performed between the two growth conditions. The *P*-value was adjusted for multiple testing using Holm’s method (Holm 1979).

### Fungal isolation and culture

For the RNA extraction, single-spore isolates were obtained from the same ascoma of *C. maritima* from Paraíso Beach following a technique described by Choi *et al.* (1999). Whereas for the quantitative reverse-transcription (RT) PCR, single-spore isolates from Pico de Oro Beach, Paraíso Beach, and Boca del Río Beach were obtained in addition to those from Paraíso Beach. Mycelia were grown at 25° in potato-dextrose liquid medium for 15 d under two salinity conditions (marine and freshwater). For the marine condition we added artificial seawater (Instant Ocean; Aquarium Systems, USA) to the medium following the instructions given by the manufacturer. Cultures were plated to check for contamination, and only uncontaminated cultures were further processed.

### RNA isolation

**Figure 1:**
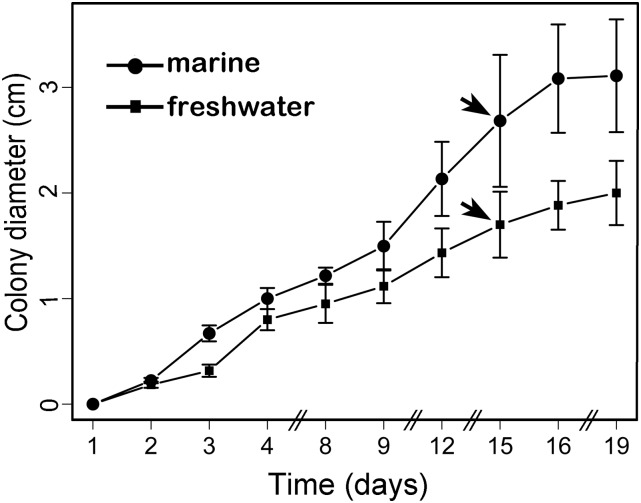
Graphical representation of *C. maritima* growth under two salinity conditions (bars represent SD). The growth in freshwater is represented by filled squares, whereas the marine growth is represented as the solid circles. Statistical significance between the curves was assessed using a permutation test to compare growth curves. The test was applied to the colony diameter until effects on the growth were most apparent, that is, 16–19 d after inoculation. The pair-wise comparisons between the fungi samples were statistically nonsignificant (*P*-value 0.0999). The arrows indicate the time of growth from where the RNA isolation was performed for either the transcriptome or the RT-qPCR analysis.

Total RNA was obtained from samples at the mid-log phase of vegetative growth (15 d) for both freshwater-grown and seawater-grown tissues (Figure 1). Frozen aliquots of mycelia were quickly ground in Trizol reagent (Invitrogen Corporation, California, USA) and RNA was subsequently isolated following the manufacturer’s instructions. The integrity of RNA was determined using the Agilent 2100 Bioanalyzer system prior to the library preparation. All the samples presented a minimum RNA Integrity Number (RIN) of eight.

### cDNA library construction and whole transcriptome sequencing

The RNA-seq libraries were prepared from total RNA using the Illumina TruSeq RNA Sample Preparation Kit following the vendor instructions. The libraries containing the cDNA from each sample were sequenced in the Illumina GAIIx platform with a sequencing configuration for 36 bp single reads.

### Bioinformatics analysis

Although some Ascomycota genomes are available, these genomes diverged early from *C. maritima* (Hedges *et al.* 2006). Therefore, to avoid evaluation bias we did not base our *de novo* transcriptome analysis on such genomes. Hence, the transcriptome of *C. maritima* was reconstructed using the Illumina reads from both growth conditions with the software Trinity (r2013-02-25) (Grabherr *et al.* 2011) with default parameters for single reads. The resulting transcripts (Supporting Information, File S1) were used as a reference to perform the differential expression (DE) analysis with no replicates and to obtain the protein products by conceptual translation using the run_Trinity_edgeR_pipeline.pl (edgeR dispersion value = 0.1) and transcripts_to_best_scoring_ORFs.pl (using TMM normalization) modules, respectively. The DE results were filtered using a False Discovery Rate (FDR) cutoff line ≤0.05 (Table S1). The GO term enrichment analysis for the DE transcripts was performed using the R package topGO using a *P*-value cutoff ≤0.05 (Alexa and Rahnenfuhrer 2010). The software Trinotate (with default parameters), Swissprot/Uniprot, and PFAM-A (Finn *et al.* 2014), all included with the Trinity package, were used for protein annotation (Table S1). Trinotate assigns to each protein, if available, the best BLASTP result from the Swissprot/Unitprot database (Uniprot Consortium 2014) and the predictions for PFAM domain, transmembrane domain, and signal peptide.

### Relative gene expression

To validate the reliability of the RNA-seq–derived expression changes between marine and freshwater *C. maritima* growth conditions, we tested the levels of seven transcripts (four preferentially expressed in marine and three preferentially detected in freshwater grown tissues) using three replicates by quantitative RT-PCR. The tested transcripts included an isopenicillin epimerase component-like protein (IPN; comp133_c0_seq1), a multipass membrane protein (MPS; comp2003_c0_seq1), an aspartic type endopeptidase (ASP-PEP; comp1743_c0_seq1), and a choline sulfatase family protein from the endoplasmic reticulum (CHS-ER; comp1764_c0_seq1). The last two transcripts presented more than a 2-fold increment in the marine growth according to the RNA-seq data but are not part of the results shown in Table S2, because they did not fulfill the established FDR < 0.05 threshold. Nonetheless, they were included in our validation experiment because of their previously reported role as osmoprotectants in microorganisms (Cregut *et al.* 2014). Their analysis also served to test the reproducibility of changes for some of the outliers close to the cutoff in our bioinformatics analysis. The freshwater increased or marine decreased transcripts included a glycosyl phosphatidyl inositol–anchored membrane protein with putative glucosidase activity (GPI-GLU; comp1325_c0_seq1), a pathogenesis-related protein with CFEM domain (PR-HYP; comp343_c0_seq1), and another stress-related protein with homology to the ferritin-like superfamily (FRT; comp1470_c0_seq1).

The RNA samples used for the quantitative RT-PCR were collected from the same growth points as those used for the RNA-seq analysis. However, the fungal isolates used for the three biological replicates were obtained from different geographic locations (see Fungal isolation and culture in the *Materials and Methods* section). Two micrograms of total RNA were treated with RQ1 DNase (Promega Corp., Madison, WI, USA) and subjected to RT using ImProm II reverse-transcriptase (Promega) according to the manufacturer’s instructions. Oligo dT was used as primer in the RT reaction. The qPCR was performed for selected genes with primers designed in the Primer3Plus Program (Table S3). The 60S ribosomal protein L16 (rpL16) was used as control gene (comp253_c0; Table S1). The amplification was performed in a 7500 DNA analyzer (Applied Biosystems, Foster, CA) using the SYBR Green PCR master mix (Applied Biosystems) for signal detection. The relative expression was calculated after normalization by the reference gene (rpL16) using the 2^−ΔΔCt^ method. The fold change was calculated with respect to the marine growth condition.

### Phylogenetic analysis

To further test the correctness of the bioinformatics analysis, especially the *de novo* transcriptome assembly and annotation of this nonmodel fungus, we compared our comp31_c0_seq1 (File S1) transcript sequence with public databases targeting for fungal orthologs. We conducted a phylogenetic analysis of the translation elongation factor 1-alpha (EF1a) sequences using the ETE2 workflow "phylomedb4" (Huerta-Cepas *et al.* 2010). Sequences of the EF1a for 77 fungal species including our transcript for *C. maritima* (comp31_c0_seq1) and *Caenorhabditis elegans* were retrieved from the accession numbers shown in Table S4. The workflow consisted of the multiple sequence alignment method using different programs: MUSCLE v3.7 (Edgar 2004), MAFFT v6.712b (Katoh *et al.* 2005), and Clustal Omega (Sievers *et al.* 2011). The alignments were performed in forward and reverse directions. The six resulting alignments were then combined with M-Coffee (Wallace *et al.* 2006). This allowed alignments to be trimmed not only based on their gap content but also based on the pairing consistency across different alignments using the program trimAl v1.2 (Capella-Gutiérrez *et al.* 2009). The resulting processed alignment was used to reconstruct a distance phylogram using Neighbor Joining (NJ) and Maximum Likelihood (ML) methods (Guindon *et al.* 2010).

## Data Availability

Data set S1 contains the *C. maritima* assembled transcript sequences. Data set S2 comprises the organismal distribution of annotated *C. maritima* transcripts. Data set S3, Data set S4, and Data set S5 encompass the expanded GO analysis for seawater and freshwater differentially expressed genes. Table S1 has the gene annotation using Trinotate (Transcriptome Functional Annotation and Analysis). Table S2 covers the differential expression analysis. Table S3 contains the list of primers used for the RT-qPCR analysis. Table S4 comprehends the list of species and EF1a accession numbers used in the phylogenetic analysis. Figure S1 shows the macroscopic and microscopic morphological characteristics of *C. maritima* isolates growing under two salinity conditions. Transcript sequencing data are available through the NCBI Sequence Read Archive under the accession number PRJNA274818, and the assembled and annotated transcripts are available through the NCBI Transcriptome Shotgun Assembly Project under the accession number GDFX00000000, and the FTP server (http://zebra.ibt.unam.mx/Corollospora_maritima_data/).

## Results

### *Corollospora maritima* growth under two physiological conditions

The early phase of growth rate of strains isolated from the Gulf of Mexico in marine and freshwater conditions was reached in 2 d, the mid-log phase was reached in 9 d, and the transition to the stationary phase was observed after 18 d of growth (Figure 1). Therefore, RNA isolation was performed at the mid-log phase of vegetative growth (15 d) for both freshwater-grown and seawater-grown tissues, because the fungal tissue at that time was sufficient for the isolation (Figure 1; Figure S1). Growth curves for both conditions exhibited similar shapes and were reproducible among the replicates tested. The statistical test computed to compare the colony diameters of *C. maritima* colonies over the course of growth did not show a significant difference between the growth rations in both growth conditions (*P*-value 0.099).

### RNA-seq sequencing and assembly

RNA-seq Illumina libraries were prepared and sequenced with the Illumina GAIIx system (see *Material and Methods*). A yield of ∼1.9 Gb of 36 bp single reads for each condition was obtained (Table 1). For the assembly process, reads from both growth conditions were pooled together to reconstruct all the transcripts expressed in both conditions. Using Trinity with default parameters (Grabherr *et al.* 2011), a total of 14,530 transcripts were obtained for a total of 16,388,241 bases. The resulting transcripts are available in File S1. For both freshwater and marine conditions, 97% of the reads corresponding to each condition were mapped back to the assembly. This result indicates that the RNA-seq assembly process incorporated almost all the reads. From the 14,530 transcripts, 14,317 protein products were predicted. Detailed statistics of the assembly and annotation are shown in Table 2. Transcript sequencing data are available through the NCBI Sequence Read Archive (SRA) under the accession number PRJNA274818 (http://www.ncbi.nlm.nih.gov/bioproject/PRJNA274818). Assembled and annotated transcripts are available through the NCBI Transcriptome Shotgun Assembly Project under the accession number GDFX00000000 and the FTP server http://zebra.ibt.unam.mx/Corollospora_maritima_data/.

**Table 1:** RNA-seq statistics

Growth Condition	Sequencing Type	Total Reads	Total Bases
Freshwater	1×36	52,664,491	1,895,921,676
Marine	1×36	52,933,400	1,905,603,111

**Table 2:** RNA-seq assembly and annotation statistics

Characteristics	Statistics
Total number of reads Total number of transcripts	105,597,891 14,530
Total number of bases	16,388,241
GC content	57.36%
Median transcript length (bp)	585
Average transcript length (bp)	1105.15
Total number of predicted proteins	14,317
Total number of proteins with annotation*^a^*	6392

a Proteins with information from any database search result or any kind of prediction were counted.

**Figure 2:**
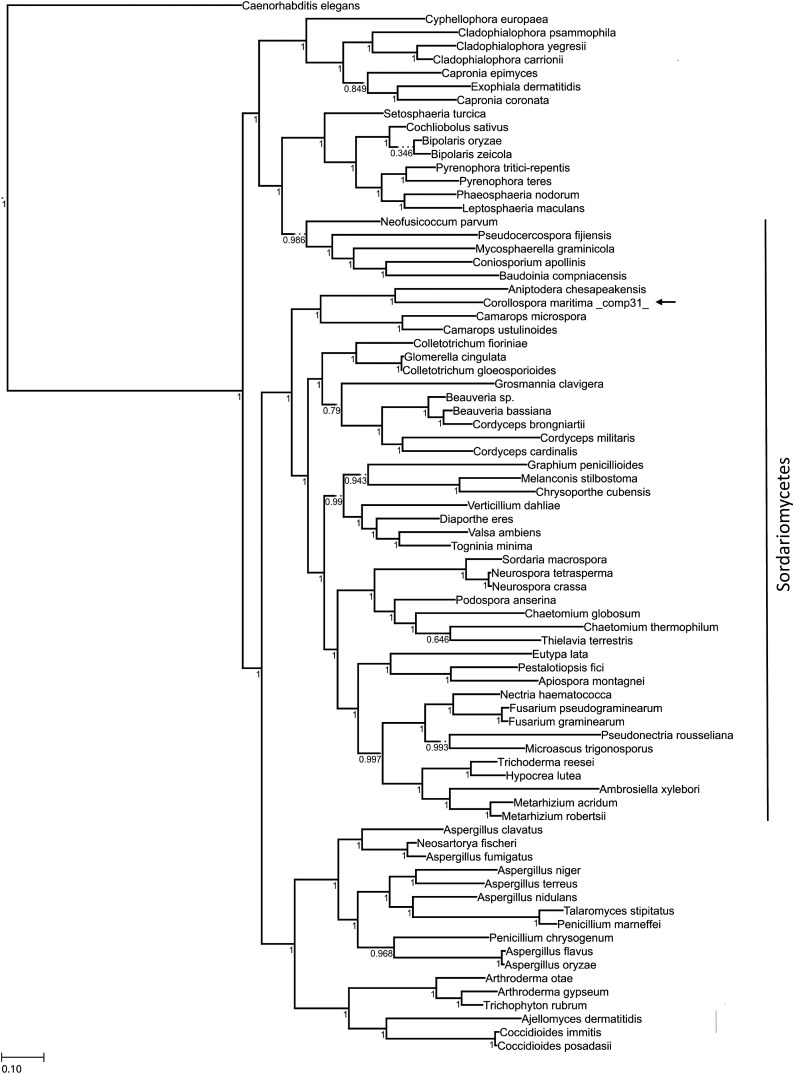
ML inferred cladogram of the EF1a nucleotide sequences alignment in 77 fungal species. Tree inferred with ETE2 under the "phylomedb4" workflow model; nodal support values represent the frequency of splits in 100 bootstrap replicates. *C. elegans* was used as an outgroup.

To predict gene functions, we used a Swissprot/Uniprot-based annotation with the best hit in BlastP available in the Trinotate package (Finn *et al.* 2014). Approximately 44% of the assembled transcripts matched to known proteins, whereas the remaining 56% had no identity to other proteins with known functions. The majority of the BlastP protein matched most to Ascomycota (73% of all). For detailed information, an interactive Krona map is available in File S2. Within Ascomycota, the sequence matches were distributed between Taphrinomycotina (all from *Schizosaccharomyces pombe*, 40%), Pezizomycotina (33%), and Saccharomycetales (27%). The phylogenetic analysis of the annotated transcript EF1a (comp31_c0_seq1) revealed that orthologs of this *C. maritima* transcript exhibit a closer relationship with the Sordariamycetes than any other fungi phylum (Figure 2).

### Differential expression analysis

For the marine and freshwater differential expression analysis, the procedure consisted of mapping the reads from each condition to the reconstructed transcripts to determine the abundance of each transcript and significant differences between conditions using the edgeR package (Robinson *et al.* 2010) through one of the pipelines (see *Materials and Methods*) included in the Trinity software. We obtained 235 differentially expressed transcripts with FDR ≤0.05, of which 103 genes were more expressed in the marine condition and 132 were more expressed in the freshwater condition (Table S2).

**Figure 3:**
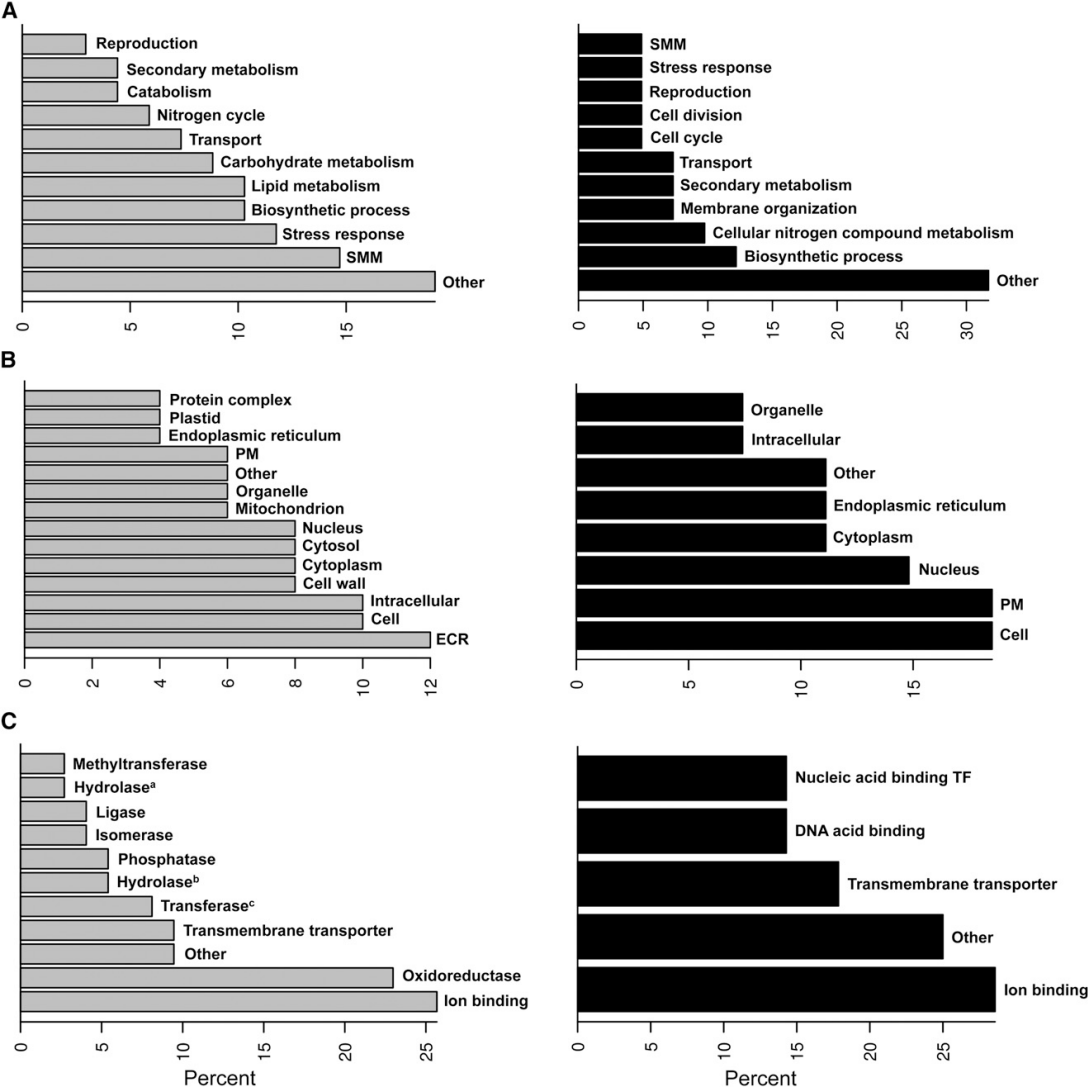
GO term distribution of marine or freshwater preferentially expressed *C. maritima* genes. The distribution is represented separately for freshwater (gray bars) and marine (black bars) upregulated genes with GO assignation in biological processes (A), cellular components (B), and molecular functions (C). “Percent” refers to the proportion (percentage) of transcripts belonging to each GO from the total of condition-enriched transcripts with GO assignation. SMM, small molecule metabolic process; PM, plasma membrane; ECR, extracellular region; TF, transcription factor; Hydrolase^a^, acting on carbon-nitrogen, but not peptide, bonds; Hydrolase^b^, acting on glycosyl bonds; Transferase^c^, transferring acyl groups.

We analyzed the growth condition-specific responses based on enrichment of Gene Ontology (GO) categories in the transcripts to discover physiological differences according to the environment. In Figure 3, slim GO categories are represented for Biological process (Figure 3A), Cellular component (Figure 3B), and Molecular function (Figure 3C). The original GO designations are available in File S3, File S4, and File S5. Approximately 69% of the differentially expressed genes were not assigned a GO because they represented hypothetical or predicted protein or showed no identity to other proteins in the database. Freshwater growth was characterized by greater expression of pathogenesis- and stress-related transcripts (Figure 3A, gray bars). Notably small molecule metabolism (SMM) was particularly enriched under this condition, whereas it was under-represented in marine growth (black bars). In addition, transcripts corresponding to the polyketide metabolism such as phenolic phthiocerol and phthiocerol biosynthesis were expressed preferentially in freshwater (File S3). Phtalide derivatives are important compounds resulting from the polyketide metabolism and have been previously described for *C. maritima* (Liberra *et al.* 1998). Hence, production of phtalides may be favored when salinity decreases in the environment of the fungus.

As expected, integral components of the membrane (PM) were overexpressed in the marine growth condition (Figure 3B, black bars), whereas transcripts stimulated in freshwater corresponded mainly to proteins enriched in extracellular (ECR) or cytoplasmic locations (Figure 3B, gray bars). Nevertheless, particular membrane components were also present in the group of freshwater-stimulated transcripts. The cellular component analysis indicated that the polyketide synthase complex is preferentially expressed in the freshwater environment (File S4). Interestingly, each growth condition was characterized by the differential expression of particular genes encoding ion-binding factors (Figure 3C). Transcripts encoding several amino acid transporters, nucleotide (FAD, NAD, NADP) binding proteins and cofactors, and proteins with oxido-reductase activity that were particularly expressed in freshwater-grown tissues of *C. maritima* belong to the classification of proteins whose molecular function is related to biotic or abiotic stress response, whereas those preferentially expressed in the marine environment include sugar or amino acid transmembrane transporters, ion channels, and transcription factors (File S5).

### Validation of specific gene expression by RT-qPCR

**Figure 4:**
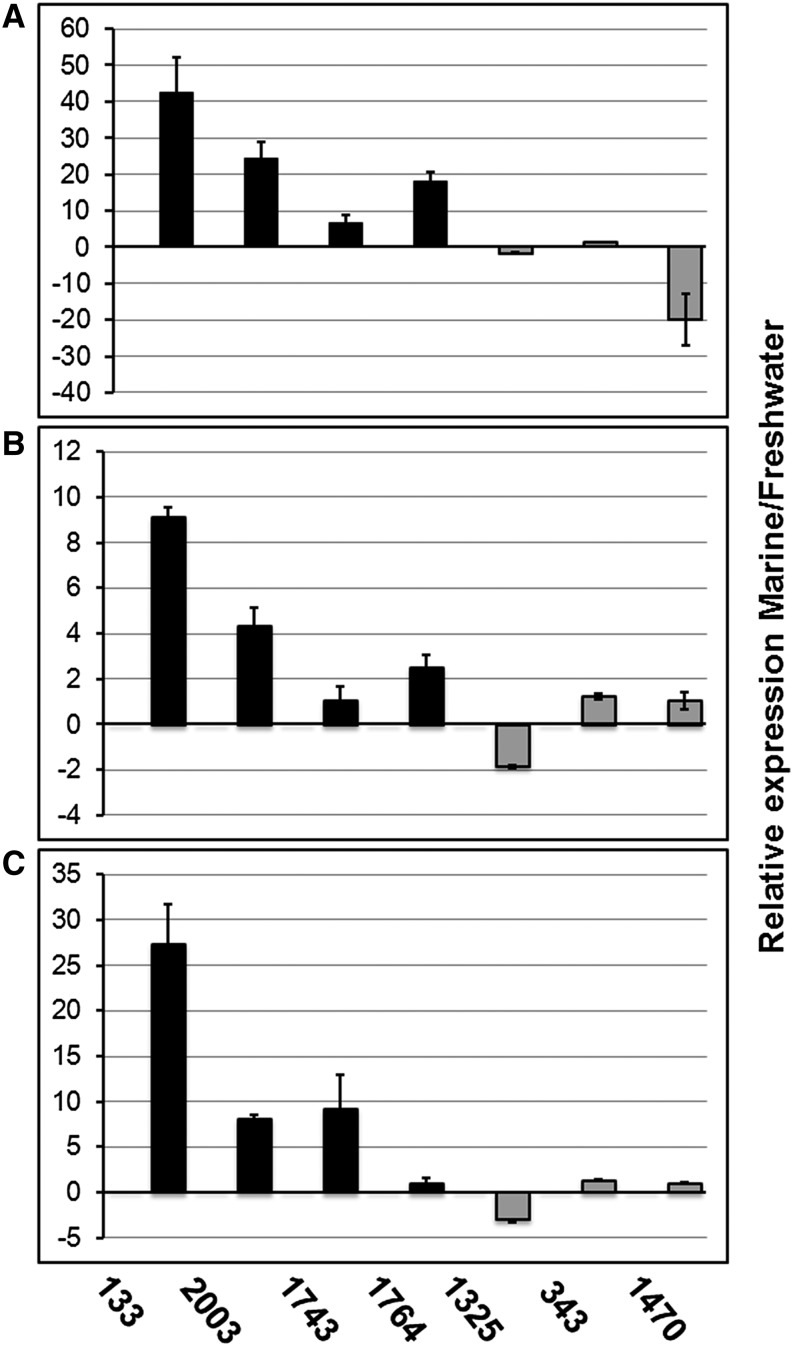
Expression analysis of selected genes during the exponential growth of *C. maritima* in freshwater and marine environments. The expression patterns of selected genes from *C. maritima* are shown as fold-change in marine compared to freshwater conditions. The expression was analyzed by RT-qPCR in three biological replicates representing fungal isolates from three different geographical locations, Pico de Oro Beach (A), Paraíso Beach (B), and Boca del Río Beach (C) at the growth point highlighted by an arrow in Figure 1. 133 (comp133_c0_seq1) is an isopenicillin epimerase component-like protein; 2003 (comp2003_c0_seq1), a multipass membrane protein; 1743 (comp1743_c0_seq1), an aspartic type endopeptidase; 1764 (comp1764_c0_seq1), a choline sulfatase family protein from the endoplasmic reticulum; 1325 (comp1325_c0_seq1), a glycosyl phosphatidyl inositol-anchored membrane protein with probable glucosidase activity; 343 (comp343_c0_seq1), a pathogenesis-related protein with CFEM domain; 1470 (comp1470_c0_seq1), a stress-related protein with homology to the ferritin-like superfamily. Black bars represent increased expression and gray bars decreased expression in a marine environment *vs.* a freshwater environment, according to the RNA-seq analysis. Error bars are shown for three technical replicates performed on each biological sample.

As observed in Figure 4, for the marine-induced transcripts (black bars) there was great consistency between replicates and with the RNA-seq results, although the relative expression ratios were generally greater in the RT-qPCR analysis. The IPN-like protein transcript or comp133_c0_seq1 consistently showed the greater induction in marine growth, regardless of the fungal isolate. Comp1743_c0_seq1 (ASP-PEP) and comp1764_c0_seq1 (CHS-ER) showed variations in the induction levels, depending on the fungal isolate. The fungal isolate corresponding to Figure 4A was the same as that used for RNA-seq (Pico de Oro Beach, Tabasco), whereas results in Figure 4B and Figure 4C corresponded to isolates from Paraíso Beach, Tabasco, and Boca del Río Beach, Veracruz, respectively.

Curiously, the freshwater-induced or marine-decreased transcripts displayed very low reproducibility as observed from the RT-qPCR analysis compared to the RNA-seq data (Figure 4; gray bars in the graphs and Table S2). Only the GPI-GLU (comp1325_c0_seq1) transcript was consistently increased in freshwater growth, whereas PR-HYP (comp343_c0_seq1) showed a behavior opposite to the RNA-seq data (Figure 4, A–C), and FRT (comp1470_c0_seq1) reproduced the freshwater-induced behavior only for the same fungal isolate (Figure 4A).

## Discussion

Even though there is a marked sampling bias due to the limited information available in databases for nonmodel fungal species, the result of the EF1a phylogenetic analysis (Figure 2) agrees with the taxonomic placement of *C. maritima*, clustering the transcript of this species (comp31_c0_seq1; File S1) within the Sordariomycetes, and showing large affinity to the *Halosphaeriaceae*. Our results also resemble the phylogeny of Ascomycota at a class level clustering the Sordariomycetes, Eurotiomycetes, and Dothideomycetes as major lineages.

Although the marine species *C. maritima* has been reported to tolerate a wide range of salinities (Byrne and Jones 1975), there was no information regarding the genetic response of this fungus to such fluctuations. In addition, genomic sequence resources are not available for this species except for conserved ribosomal genes used in biodiversity characterization. Therefore, using an RNA-seq approach we identified *C. maritima* candidate genes upregulated in marine or freshwater growth. Because the lack of replication would affect the statistical results, we considered the results as a guide to explore the probable DE genes. The most appealing and interesting genes in terms of differential expression results and annotation were validated in the present work by quantitative RT-PCR. The identification of gene candidates possibly regulated by fluctuations in the marine environment of this species represent a glimpse of the mechanisms underlying osmotic homeostasis in saltwater and a molecular basis for its physiological adaptation not only to inhabit marine sandy beaches but also to represent one of the most abundant species in many geographical locations (Velez *et al.* 2013).

Fungal cell wall is a structure with high plasticity that protects the cell from environmental stresses including changes in osmotic pressure. The fungal cell wall has a unique and complex structure containing glucan, chitin, and glycoproteins (Bowman and Free 2006). Some studies have demonstrated that changes in cell wall may occur in response to environmental stress (Fuchs and Mylonakis 2009). However, no information is available regarding the proteins and enzymes that are necessary for the control of cell wall organization in marine fungi during stress circumstances. Our results revealed several genes that are potentially involved in the modification and biosynthesis of cell wall to resist osmotic changes such as the GPI-anchored putative glucosidase (GPI-GLU; comp1325_c0_seq1) and the aspartic-type endopeptidase (ASP-PEP; comp1743_c0_seq1).

GPI-GLU has been associated with the glycoside hydrolase family 16 (PFAM 00722), with anchored components of the membrane (GO:0031225), and with cellular components of the cell wall and membrane (GO: 0005618 and GO: 0005886). Moreover, these types of proteins have been reported to be widely distributed in the fungal kingdom targeting β-glycans on fungal cell wall, and to be involved in fungal cell wall reinforcement and biosynthesis (Adams 2004; Kawai *et al.* 2006). ASP-PEP, associated with the aspartic-type signal peptidase activity (PFAM 00026 and GO: 0004190), are predicted to be anchored to the cellular membrane (GO:0031225 and GO:0005886) and were upregulated in saltwater conditions. The function of fungal secreted proteases varies, yet it has been widely described that for pathogenic fungi, proteases are important for the virulence, the adherence process, penetration of tissues, and in interactions with the immune system of the infected host (Monod *et al.* 2002). Moreover, many fungal species secrete proteases when grown in a medium containing protein as the sole nitrogen source (Brouta *et al.* 2001; Togni *et al.* 1991). However, because *C. maritima* is a saprobiotic fungus, which was grown in media made from potato infusion and dextrose not containing protein, it is improbable that aspartic peptidases play such roles. Instead, they may be involved in cell-wall assembly and/or remodeling. Studies performed on yeasts have confirmed the importance of aspartic peptidases for cell-wall integrity. So, the high occurrence of the ASP-PEP transcripts might be related to the cellular membrane metabolism linked to periods of active cell-wall synthesis (Gagnon-Arsenault *et al.* 2006). These results agree with previous observations of the growing dynamics of this ascomycete in saltwater conditions, which are more vigorous than in freshwater, indicating this fungus is well-suited to inhabit marine environments with effective proliferation.

An interesting transcript upregulated in freshwater was comp343_c0_seq1 corresponding to a PR-like protein, with the fungal-specific domain CFEM (PFAM 05730), found in extracellular membranes (GO:0005618, GO:0005576, GO:0016020). Such a domain has been proposed to play an important role in cell-surface receptors, as signal transducers, and as adhesion molecules in host–pathogen interactions (Kulkarni *et al.* 2003). Because *C. maritima* is a saprobic species, it is unlikely this domain plays a role in pathogenesis. Instead it might serve as a characteristic signature for a subset of proteins that function in the extracellular environment signaling osmotic changes. However, the behavior of this transcript did not reproduce the freshwater increase initially detected in RNA-seq experiments. Instead, we found it by using RT-qPCR; it was well-expressed in both freshwater and marine environments, as opposed to GPI-GLU, which was preferentially expressed in freshwater (Figure 4). This indicates that the signaling performed by this PR-like protein is probably relevant for the fungal growth in either environment.

The upregulation of FER transcripts corresponding to a FER protein with ferritin-like domain (PFAM 13668; comp1470_c0_seq1) in freshwater was an interesting finding, because proteins carrying this domain (GO: 0033554) have been reported to play an important role in fungal responses to external stress (Ludin *et al.* 1995). Although *C. maritima* is able to grow in freshwater conditions, the upregulation of this gene suggests that the condition could represent a hostile state for the unchaining stress-response signaling pathways of this marine fungus. Interestingly, the freshwater upregulation of FER was not reproduced in all the replicates, suggesting that the fungal response to freshwater might be associated with local adaptation mechanisms. Local adaptation results when populations evolve in response to geographically variable selection. Several studies have recognized that local adaptation is extremely common and facilitates species range expansion (Hereford 2009). We analyzed isolates of a cosmopolitan marine fungus from diverse beaches in the Gulf of Mexico exhibiting differential biotic and abiotic characteristics (*i.e.*, freshwater input and species composition); therefore, local adaptation signals are a reasonable result. The freshwater upregulation of FER was reproduced in isolates from beaches where *C. maritima* represents the most abundant species (beaches of Paraíso and Boca del Río), confronting little competition. In contrast, in Pico de Oro Beach, *C. maritima* was the second most abundant species coexisting with the dominant species and, hence, facing higher competition (Velez *et al.* 2013, 2015). These variations on fungal community structure and abiotic characteristics among beaches might lead to the physiological variation of *C. maritima* through local adaptation mechanisms, resulting in differential gene expression among isolates (Tiffin and Ross-Ibarra 2014).

Contrary to the variable behavior of selected freshwater upregulated candidate genes, those representing genes preferentially expressed in marine environment showed consistent patterns for *C. maritima* isolates from different beaches. This supports their role in the fungal adaptation to marine environment. The expression of an endoplasmic reticulum transmembrane protein, MPS (comp2003_c0_seq1), was stimulated 4-fold to 25-fold in saltwater condition. MPS is perhaps involved in the maintenance of osmotic homeostasis of this ascomycete in marine environments. By sequence homology, MPS is related to mechanosensitive ion channels (PFAM 00924 and GO: 0008381) responsible of the endoplasmic reticulum (GO:0005783 and GO:0005789) membrane transport (GO:0016021). Ion transporters and their regulatory systems fulfill several key physiological functions in providing optimal intracellular ion concentrations for several systems (Serrano *et al.* 1999). Mechanosensitive ion channel molecules have been reported to play an important role in the ion homeostasis during salt stress in yeasts (Chen *et al.* 2003). Therefore, this protein might play a decisive part in the adaptation to osmotic stress, chiefly in the management of osmolarity transitions due to salt fluctuations in the environment.

Likewise, the expression of an IPN-like gene (comp133) was 9-fold to 40-fold over-represented in saltwater as compared to freshwater. The correspondent protein is linked to molecules with ligase activity widely reported for fungal genomes (GO: 0016874); specifically, AMP binding enzymes (PFAM 00501 and PFAM 13193) are associated with isopenicillin-N epimerase activity and the penicillin biosynthetic process (GO: 0045439 and GO: 0042318). However, the upregulation of this gene in the marine growth condition could be a result of the secondary *C. maritima* metabolism. These metabolites are generally helpful for the fungus, but they are not necessary for survival, and their production is presumably costly to maintain. However, for some reason *C. maritima* maintains a high expression of this gene in saltwater, possibly due to a different unknown function of the protein or as a result of its adaptation to the marine environment.

Finally, the upregulation of a transcript encoding a choline sulfatase family protein (PFAM 00884 and 12411, GO: 0047753 and 0042425) in saltwater might be related to the osmotic regulation and marine adaptation of *C. maritima*. It has been reported that these enzymes might perform two putative roles in microorganisms: as osmoprotectants or as reservoir of C, N, and S. However, no global feature between these two distinct activities has been clarified among the whole microbial diversity (Cregut *et al.* 2014). Feasibly, an optimal maintenance of the *C. maritima* metabolism under high-salt conditions could be achieved at least partially due to a higher expression of this gene.

In conclusion, we report a first exploration of *C. maritima* gene expression during exponential growth under two contrasting conditions: marine and freshwater. Despite the lack of biological replicates, the high sequencing depth and the orthogonal validation using RT-qPCR allowed us to corroborate some of the genes reported as differentially expressed in both conditions. In addition, several of the genes reported as differentially expressed failed to detect a significant match to a protein in the database with a functional annotation. These unannotated proteins will be the subject of further characterization to elucidate their role in fungal biology. The consistently higher expression of selected genes in the marine growth condition is likely indicative of their role as osmoprotectants during the adaptation of this species to the saline environment, although, as mentioned before, further research aiming to explore the functional relevance of the corresponding proteins is required. Conversely, a greater amount of genes appear upregulated in freshwater, but the variability of their expression levels in independent biological replicates as measured by RT-qPCR suggests that their role in salinity fluctuations is uncertain or could be highly dependent on the original environment of the species. The data presented here also provide the annotation of transcripts for *C. maritima* for the first time and a portfolio of candidate genes for further studies of the evolution and adaptation of marine ascomycetes.

## 

## Supplementary Material

Supporting Information
