# Supplementary material for: Comparative Transcriptome Analysis of the Cosmopolitan Marine Fungus Corollospora maritima Under Two Physiological Conditions
Source: G3 (Bethesda). 2015 Jun 26;5(9):1805–14. doi: 10.1534/g3.115.019620 (PMC4555217; doi:10.1534/g3.115.019620)
Supplement: Supporting Information [file supp_5_9_1805__index.html]

Comparative Transcriptome Analysis of the Cosmopolitan Marine Fungus Corollospora maritima Under Two Physiological Conditions — Supporting Information 

# Comparative Transcriptome Analysis of the Cosmopolitan Marine Fungus *Corollospora maritima* Under Two Physiological Conditions

## Supporting Information for Velez *et al.*, 2015

**Files in this Data Supplement:**

- Supporting Information - Figure S1, Tables S1-S4, and Files S1-S5 (PDF, 558 KB)
- Figure S1 - Macroscopic and microscopic morphological characteristics of *C. maritima* isolates growing under two salinity conditions. (PDF, 200 KB)
- Table S3 - List of primers used for the RT-qPCR analysis. (PDF, 137 KB)
- Table S4 - List of species used in the phylogenetic analysis. (PDF, 141 KB)
- File S3 - Expanded GO analysis for seawater and freshwater differentially expressed genes. (PDF, 139 KB)
- File S4 - Expanded GO analysis for seawater and freshwater differentially expressed genes. (PDF, 43 KB)
- File S5 - Expanded GO analysis for seawater and freshwater differentially expressed genes. (PDF, 143 KB)
- Table S1 - Gene annotation using Trinotate (Transcriptome Functional Annotation and Analysis). (.zip, 1 MB)
- Table S2 - Differential expression analysis. (.zip, 55 KB)
- File S1 - *Corollospora maritima* assembled transcript sequences. (.zip, 5 MB)
- File S2 - Organismal distribution of annotated *Corollospora maritima* transcripts. (.zip, 10 KB)
