# Supplementary material for: Comparative Transcriptome Analysis of the Cosmopolitan Marine Fungus Corollospora maritima Under Two Physiological Conditions
Source: G3 (Bethesda). 2015 Jun 26;5(9):1805–14. doi: 10.1534/g3.115.019620 (PMC4555217; doi:10.1534/g3.115.019620)

G3·Genes | Genomes | Genetics

Comparative transcriptome analysis of the cosmopolitan marine fungus *Corollospora maritima* under two physiological conditions

Patricia Velez,[*,1] Naholi D. Alejandri-Ramírez,[§] María C. González,[*] Karel J. Estrada,[†] Alejandro Sanchez-Flores,[†] Tzvetanka D. Dinkova[§]

[*] Departamento de Botánica, Instituto de Biología, Universidad Nacional Autónoma de México, Distrito Federal, México, 04510
[§] Departamento de Bioquímica, Facultad de Química, Universidad Nacional Autónoma de México, Distrito Federal, México, 04510
[†] Unidad Universitaria de Apoyo Bioinformático, Instituto de Biotecnología, Universidad Nacional Autónoma de México, Cuernavaca, Morelos, México, 62210
[1]Departamento de Ecología Evolutiva, Instituto de Ecología, Universidad Nacional Autónoma de México, Distrito Federal, México, 04510

Data deposition: Transcript sequencing data are available through the NCBI Sequence Read Archive under the accession number PRJNA274818, and the assembled and annotated transcripts are available through the NCBI Transcriptome Shotgun Assembly Project under the accession number GDFX00000000, and the FTP server http://zebra.ibt.unam.mx/Corollospora_maritima_data/

Corresponding authors
Patricia Velez, Departamento de Ecología Evolutiva, Instituto de Ecología, Circuito exterior s/n, Ciudad Universitaria, Copilco, Coyoacán, Apartado Postal 70-197 México, Distrito Federal C.P. 04510, telephone +52 55 56229006, email address: pvelezaguilar@gmail.com

Tzvetanka D. Dinkova, Departamento de Bioquímica, Facultad de Química, Circuito exterior s/n, Ciudad Universitaria, Copilco, Coyoacán, Apartado Postal 70-275 México, Distrito Federal C.P. 04510, telephone +52 55 56225277, email address: cesy@unam.mx

DOI: 10.1534/g3.115.019620

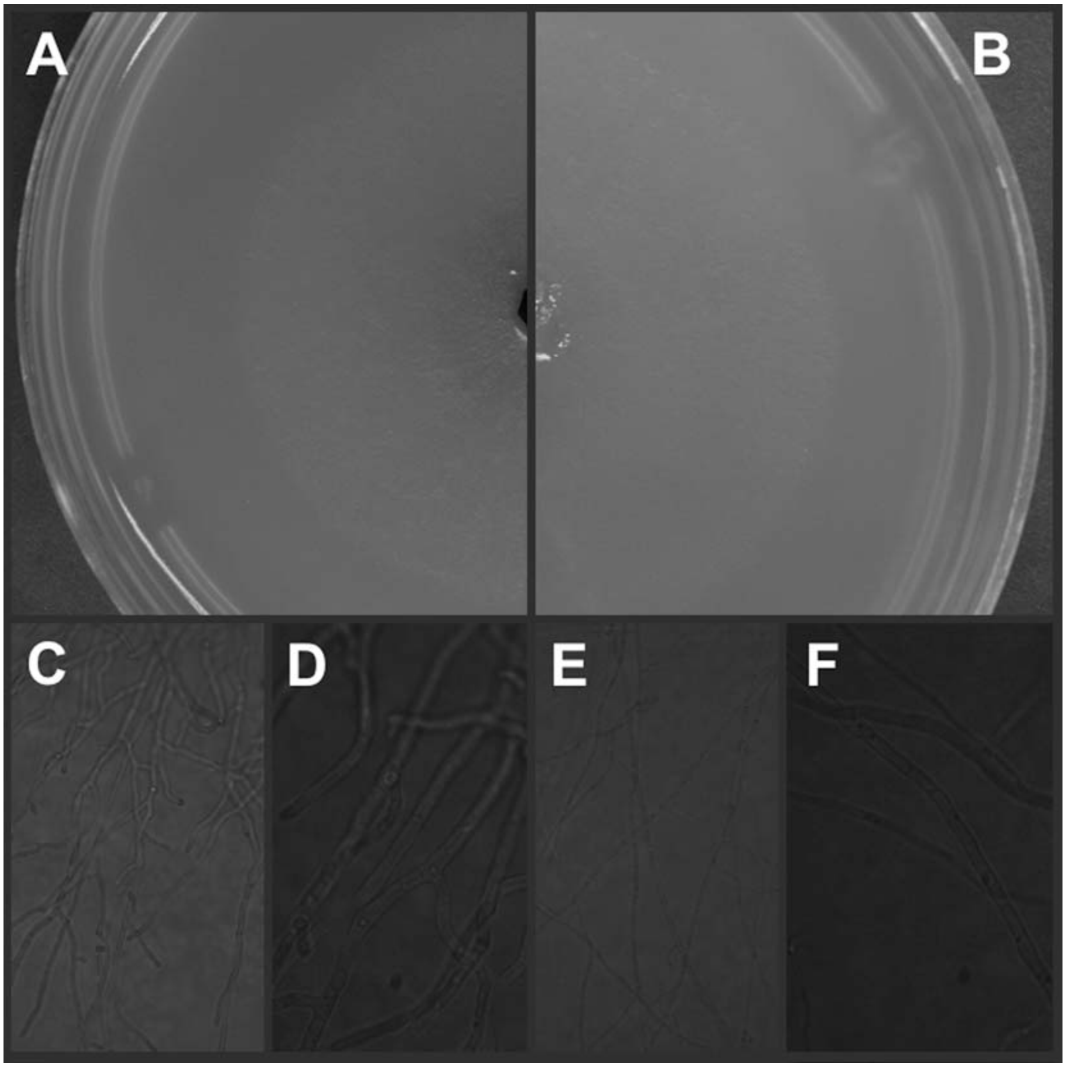

Figure S1 Macroscopic and microscopic morphological characteristics of *C. maritima* isolates growing under two salinity conditions. A, C, and D isolates growing under marine condition. B, E, and F isolates growing under freshwater condition. Scale bars: C and E = 30 µm, D and F = 5 µm.

Tables S1-S2

Available for download at www.g3journal.org/lookup/suppl/doi:10.1534/g3.115.019620/-/DC1

Table S1 Gene annotation using Trinotate (Transcriptome Functional Annotation and Analysis). The table shows for each gene its possible annotation (if available).

Top_BLASTX_hit = Best BLASTP result from the Swissprot/Unitprot database, predictions for PFAM domain, transmembrane domain, and signal peptide.

Table S2 Differential expression analysis. The table shows the values from main EdgeR statistic for each gene. id = Gene Id, logFC = log2-fold-change (a measure describing how much the transcript quantity changes between the two conditions), logCPM = log2-counts-per-million (used to normalize the counts), PValue = Function of the observed sample results (a statistic). A *P*-value equal to or smaller than the significance level (thresholds), it suggests that the result is accepted as true. FDR = False discovery rate (FDR procedures are designed to control the expected proportion of incorrectly results "false discoveries").

Table S3 List of primers used for the RT-qPCR analysis.

Primer ID	Sequence	Product size (bp)
1325-F	5′-CTTTTCCCTTCCCCATCCCC-3′	108
1325-R	5′-TAACGAGTGTGGTGGAAGCC-3′	
343-F	5′-GCTTTGGGCCTCTCGAGAAT-3′	122
343-R	5′-GCCCGCACAAACAAGTCAAT-3′	
1470-F	5′-TACGGGAAACAGTGGACACG-3′	149
1470-R	5′-ATTCGCCATCAGATCGCGAT-3′	
133-F	5′-CTTTCCGGTCGTCACCTGAA-3′	86
133-R	5′-GAGCAGAGTGAGGAGGGAGA-3′	
2003-F	5′-GAGAACGATGGCGACGATCT-3′	119
2003-R	5′-GACTCGGCGATCCATTGGAT-3′	
1743-F	5′-CCAGGGACGAGAAGAGGAGA-3′	97
1743-R	5′-GATCCATTCAAACCGCGTCG-3′	
1764-F	5′-ACCCCAACTCGCAGATCAAG-3′	145
1764-R	5′-GCTCCGATCTTCATGGGGAG-3′	
253-F (HK)	5′-CAAGAGGAACCCAATCAAGC-3′	121
253-R (HK)	5′-CCGCACCAAATATCTCAACC-3′	

Primer ID: the number refers to the identification designed to the sequence assembled from the RNA-seq analysis (i.e. comp1325_c0_seq1 in Table S1); F: forward; R: reverse; HK: housekeeping (the selection was made based on little or no change in the expression levels between marine and freshwater growth conditions according to the RNA-seq analysis)

Table S4 List of species used in the phylogenetic analysis.

EF1a GI number	Taxon
261193965	*Ajellomyces dermatitidis*
94411556	*Ambrosiella xylebori*
94411514	*Aniptodera chesapeakensis*
112785275	*Apiospora montagnei*
315055070	*Arthroderma gypseum*
296825909	*Arthroderma otae*
121702562	*Aspergillus clavatus*
238499326	*Aspergillus flavus*
146322500	*Aspergillus fumigatus*
67527948	*Aspergillus nidulans*
317037312	*Aspergillus niger*
317150172	*Aspergillus oryzae*
115389365	*Aspergillus terreus*
627796928	*Baudoinia compniacensis*
667647750	*Beauveria bassiana*
152937527	*Beauveria sp.*
627920131	*Bipolaris oryzae*
628208859	*Bipolaris zeicola*
392926564	*Caenorhabditis elegans*
760173891	*Camarops microspora*
94411500	*Camarops ustulinoides*
628249696	*Capronia coronata*
628255550	*Capronia epimyces*
116193652	*Chaetomium globosum*
576035729	*Chaetomium thermophilum*
114150187	*Chrysoporthe cubensis*
671164360	*Cladophialophora carrionii*
628333805	*Cladophialophora psammophila*
628278561	*Cladophialophora yegresii*
14150842	*Coccidioides immitis*
303317099	*Coccidioides posadasii*
628083431	*Cochliobolus sativus*
615455227	*Colletotrichum fioriniae*
596702914	*Colletotrichum gloeosporioides*
667829377	*Coniosporium apollinis*
152937537	*Cordyceps brongniartii*

109628446	*Cordyceps cardinalis*
573976171	*Cordyceps militaris*
671145855	*Cyphellophora europaea*
94411394	*Diaporthe eres*
629675377	*Eutypa lata*
684162144	*Exophiala dermatitidis*
758195067	*Fusarium graminearum*
685872678	*Fusarium pseudograminearum*
27960766	*Glomerella cingulata*
760173893	*Graphium penicillioides*
724472930	*Grosmannia clavigera*
27960782	*Hypocrea lutea*
396461041	*Leptosphaeria maculans*
110810461	*Melanconis stilbostoma*
629684016	*Metarhizium acridum*
761845151	*Metarhizium robertsii*
94411527	*Microascus trigonosporus*
398399249	*Mycosphaerella graminicola*
302921063	*Nectria haematococca*
615398955	*Neofusicoccum parvum*
119496508	*Neosartorya fischeri*
758979797	*Neurospora crassa*
698996823	*Neurospora tetrasperma*
255935410	*Penicillium chrysogenum*
212527771	*Penicillium marneffei*
630011474	*Pestalotiopsis fici*
169616974	*Phaeosphaeria nodorum*
171684990	*Podospora anserina*
631373337	*Pseudocercospora fijiensis*
27960780	*Pseudonectria rousseliana*
330917620	*Pyrenophora teres*
189188583	*Pyrenophora tritici-repentis*
636581076	*Setosphaeria turcica*
336264741	*Sordaria macrospora*
242784620	*Talaromyces stipitatus*
367042017	*Thielavia terrestris*
631242603	*Togninia minima*
589104066	*Trichoderma reesei*
21540003	*Trichophyton rubrum*

114150196	*Valsa ambiens*
697073754	*Verticillium dahliae*
comp31*	*Corollospora maritima*

*This sequence corresponds to the transcriptome reported in this work

Files S1-S2

Available for download at www.g3journal.org/lookup/suppl/doi:10.1534/g3.115.019620/-/DC1

File S1 *Corollospora maritima* assembled transcript sequences.

File S2 Organismal distribution of annotated *Corollospora maritima* transcripts.

Files S3-S5

Expanded GO analysis for seawater and freshwater differentially expressed genes.

The numbers at the bottom refer to the number of transcripts from each GO classification enriched in freshwater (grey bars) or marine (black bars) environment. Only significantly enriched transcripts were considered.

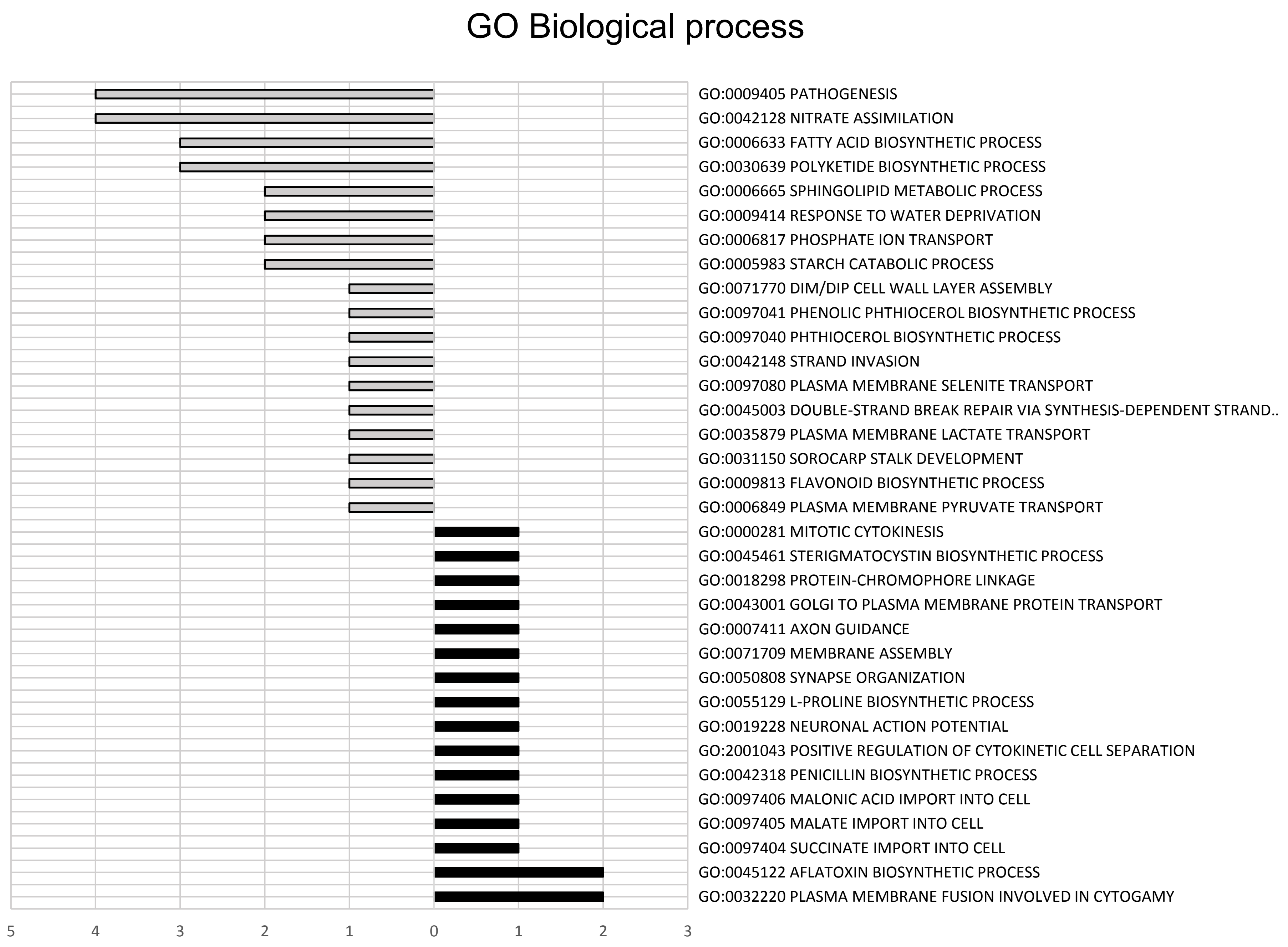
GO Biological process
GO:0009405 PATHOGENESIS
GO:0042128 NITRATE ASSIMILATION
GO:0006633 FATTY ACID BIOSYNTHETIC PROCESS
GO:0030639 POLYKETIDE BIOSYNTHETIC PROCESS
GO:0006665 SPHINGOLIPID METABOLIC PROCESS
GO:0009414 RESPONSE TO WATER DEPRIVATION
GO:0006817 PHOSPHATE ION TRANSPORT
GO:0005983 STARCH CATABOLIC PROCESS
GO:0071770 DIM/DIP CELL WALL LAYER ASSEMBLY
GO:0097041 PHENOLIC PHTHIOCEROL BIOSYNTHETIC PROCESS
GO:0097040 PHTHIOCEROL BIOSYNTHETIC PROCESS
GO:0042148 STRAND INVASION
GO:0097080 PLASMA MEMBRANE SELENITE TRANSPORT
GO:0045003 DOUBLE-STRAND BREAK REPAIR VIA SYNTHESIS-DEPENDENT STRAND..
GO:0035879 PLASMA MEMBRANE LACTATE TRANSPORT
GO:0031150 SOROCARP STALK DEVELOPMENT
GO:0009813 FLAVONOID BIOSYNTHETIC PROCESS
GO:0006849 PLASMA MEMBRANE PYRUVATE TRANSPORT
GO:0000281 MITOTIC CYTOKINESIS
GO:0045461 STERIGMATOCYSTIN BIOSYNTHETIC PROCESS
GO:0018298 PROTEIN-CHROMOPHORE LINKAGE
GO:0043001 GOLGI TO PLASMA MEMBRANE PROTEIN TRANSPORT
GO:0007411 AXON GUIDANCE
GO:0071709 MEMBRANE ASSEMBLY
GO:0050808 SYNAPSE ORGANIZATION
GO:0055129 L-PROLINE BIOSYNTHETIC PROCESS
GO:0019228 NEURONAL ACTION POTENTIAL
GO:2001043 POSITIVE REGULATION OF CYTOKINETIC CELL SEPARATION
GO:0042318 PENICILLIN BIOSYNTHETIC PROCESS
GO:0097406 MALONIC ACID IMPORT INTO CELL
GO:0097405 MALATE IMPORT INTO CELL
GO:0097404 SUCCINATE IMPORT INTO CELL
GO:0045122 AFLATOXIN BIOSYNTHETIC PROCESS
GO:0032220 PLASMA MEMBRANE FUSION INVOLVED IN CYTOGAMY
5
4
3
2
1
0
1
2
3

GO Cellular component

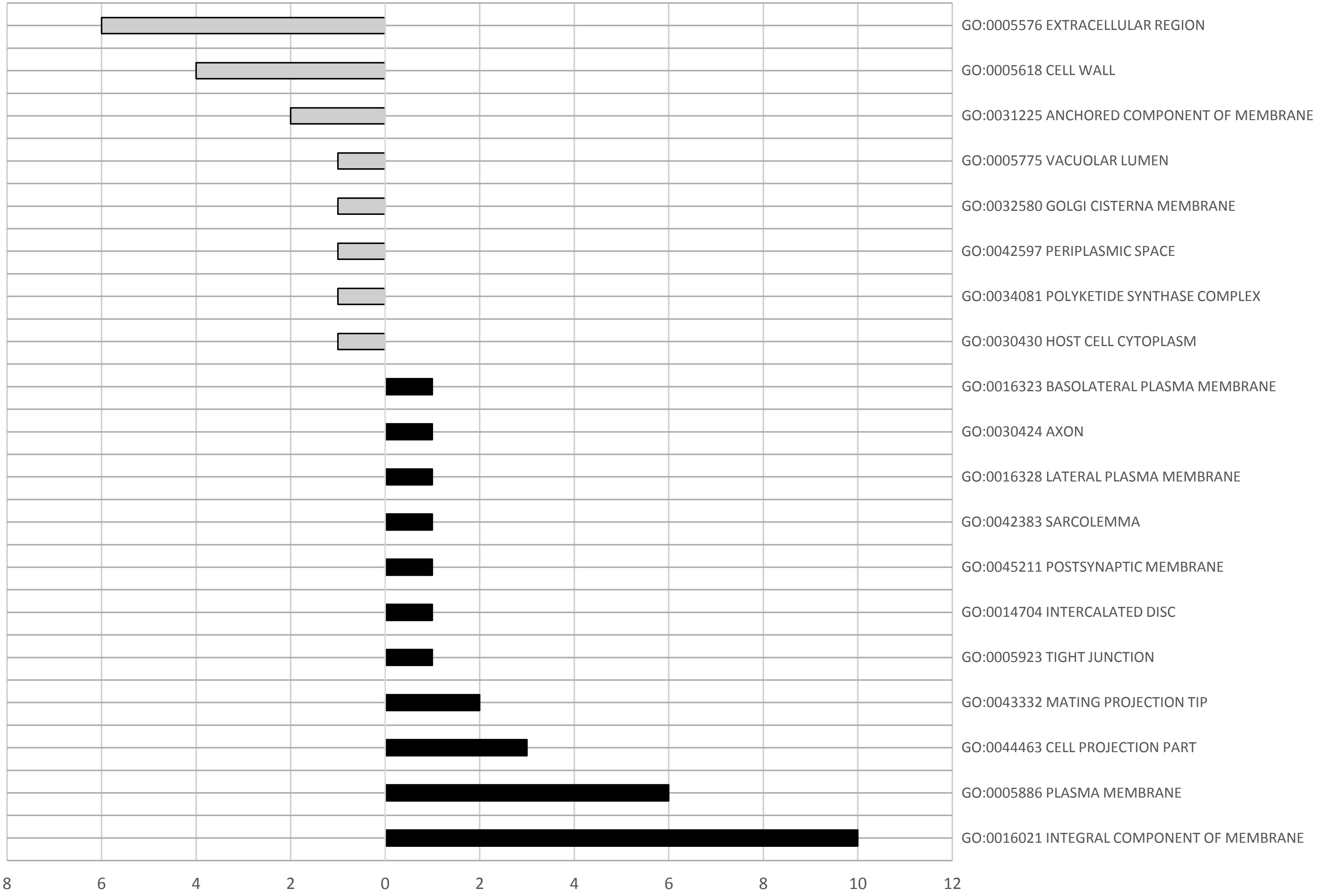

GO Molecular function

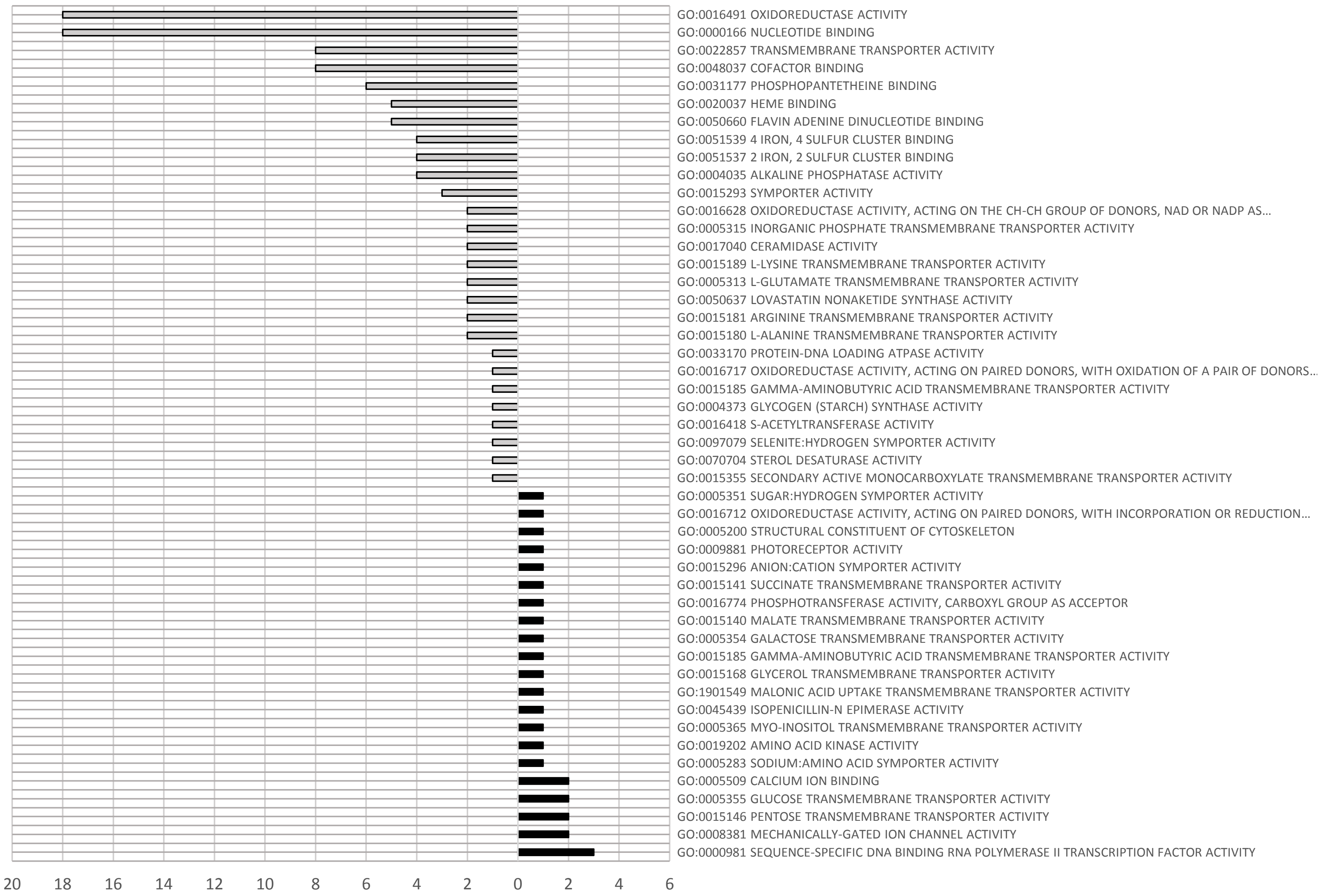

Supplement: Supporting Information [file supp_g3.115.019620_019620SI.pdf]
