# Supplementary material for: Comparative Transcriptome Analysis of the Cosmopolitan Marine Fungus Corollospora maritima Under Two Physiological Conditions
Source: G3 (Bethesda). 2015 Jun 26;5(9):1805–14. doi: 10.1534/g3.115.019620 (PMC4555217; doi:10.1534/g3.115.019620)
Supplement: Supporting Information [file supp_g3.115.019620_FigureS1.pdf]

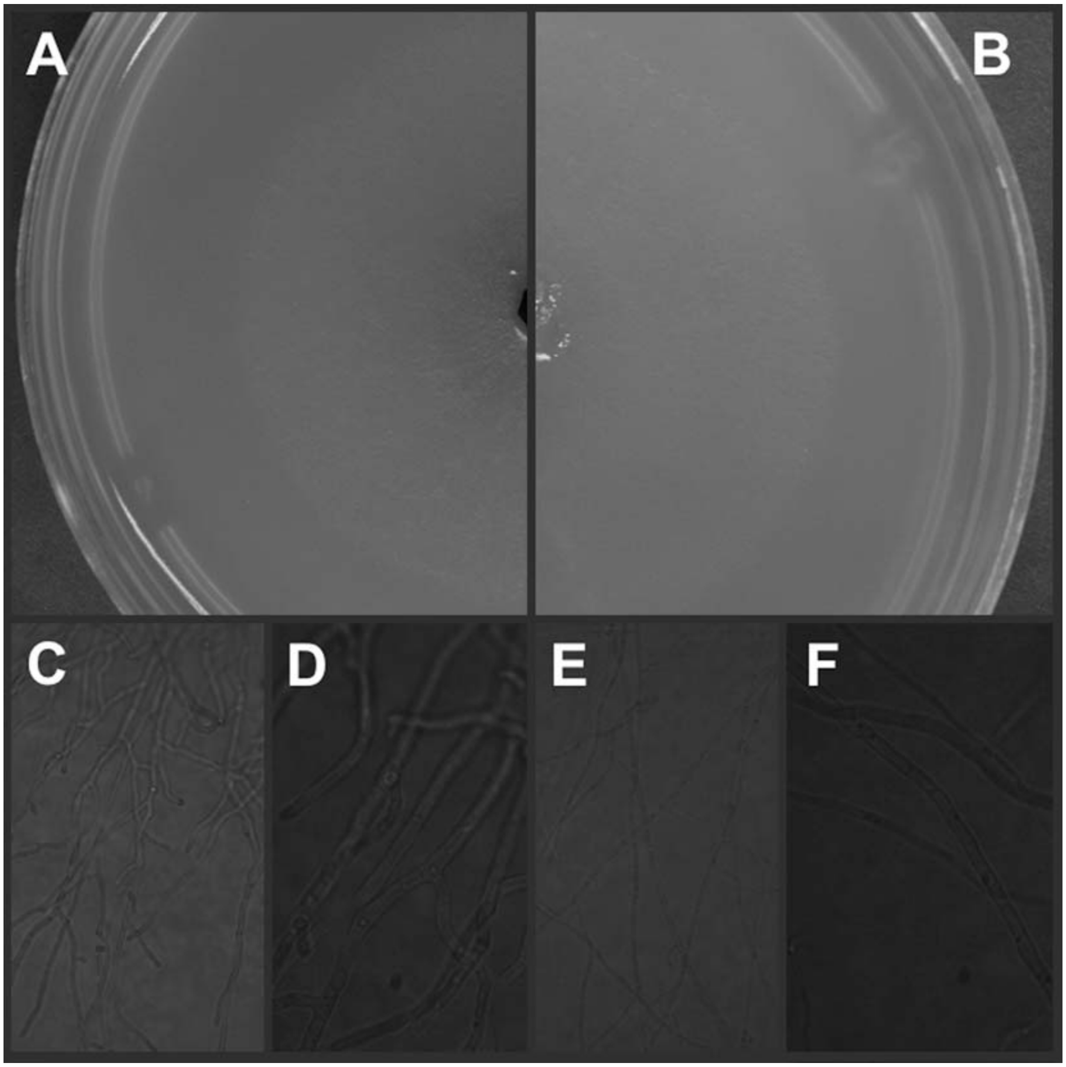

Figure S1 Macroscopic and microscopic morphological characteristics of *C. maritima* isolates growing under two salinity conditions. A, C, and D isolates growing under marine condition. B, E, and F isolates growing under freshwater condition. Scale bars: C and E = 30 µm, D and F = 5 µm.
