# Supplementary material for: Comparative Transcriptome Analysis of the Cosmopolitan Marine Fungus Corollospora maritima Under Two Physiological Conditions
Source: G3 (Bethesda). 2015 Jun 26;5(9):1805–14. doi: 10.1534/g3.115.019620 (PMC4555217; doi:10.1534/g3.115.019620)
Supplement: Supporting Information [file supp_g3.115.019620_TableS3.pdf]

Table S3 List of primers used for the RT-qPCR analysis.

Primer ID	Sequence	Product size (bp)
1325-F	5′-CTTTTCCCTTCCCCATCCCC-3′	108
1325-R	5′-TAACGAGTGTGGTGGAAGCC-3′	
343-F	5′-GCTTTGGGCCTCTCGAGAAT-3′	122
343-R	5′-GCCCGCACAAACAAGTCAAT-3′	
1470-F	5′-TACGGGAAACAGTGGACACG-3′	149
1470-R	5′-ATTCGCCATCAGATCGCGAT-3′	
133-F	5′-CTTTCCGGTCGTCACCTGAA-3′	86
133-R	5′-GAGCAGAGTGAGGAGGGAGA-3′	
2003-F	5′-GAGAACGATGGCGACGATCT-3′	119
2003-R	5′-GACTCGGCGATCCATTGGAT-3′	
1743-F	5′-CCAGGGACGAGAAGAGGAGA-3′	97
1743-R	5′-GATCCATTCAAACCGCGTCG-3′	
1764-F	5′-ACCCCAACTCGCAGATCAAG-3′	145
1764-R	5′-GCTCCGATCTTCATGGGGAG-3′	
253-F (HK)	5′-CAAGAGGAACCCAATCAAGC-3′	121
253-R (HK)	5′-CCGCACCAAATATCTCAACC-3′	

Primer ID: the number refers to the identification designed to the sequence assembled from the RNA-seq analysis (i.e. comp1325_c0_seq1 in Table S1); F: forward; R: reverse; HK: housekeeping (the selection was made based on little or no change in the expression levels between marine and freshwater growth conditions according to the RNA-seq analysis)
