# Supplementary material for: Comparative Transcriptome Analysis of the Cosmopolitan Marine Fungus Corollospora maritima Under Two Physiological Conditions
Source: G3 (Bethesda). 2015 Jun 26;5(9):1805–14. doi: 10.1534/g3.115.019620 (PMC4555217; doi:10.1534/g3.115.019620)
Supplement: Supporting Information [file supp_g3.115.019620_TableS4.pdf]

Table S4 List of species used in the phylogenetic analysis.

EF1a GI number	Taxon
261193965	*Ajellomyces dermatitidis*
94411556	*Ambrosiella xylebori*
94411514	*Aniptodera chesapeakensis*
112785275	*Apiospora montagnei*
315055070	*Arthroderma gypseum*
296825909	*Arthroderma otae*
121702562	*Aspergillus clavatus*
238499326	*Aspergillus flavus*
146322500	*Aspergillus fumigatus*
67527948	*Aspergillus nidulans*
317037312	*Aspergillus niger*
317150172	*Aspergillus oryzae*
115389365	*Aspergillus terreus*
627796928	*Baudoinia compniacensis*
667647750	*Beauveria bassiana*
152937527	*Beauveria sp.*
627920131	*Bipolaris oryzae*
628208859	*Bipolaris zeicola*
392926564	*Caenorhabditis elegans*
760173891	*Camarops microspora*
94411500	*Camarops ustulinoides*
628249696	*Capronia coronata*
628255550	*Capronia epimyces*
116193652	*Chaetomium globosum*
576035729	*Chaetomium thermophilum*
114150187	*Chrysoporthe cubensis*
671164360	*Cladophialophora carrionii*
628333805	*Cladophialophora psammophila*
628278561	*Cladophialophora yegresii*
14150842	*Coccidioides immitis*
303317099	*Coccidioides posadasii*
628083431	*Cochliobolus sativus*
615455227	*Colletotrichum fioriniae*
596702914	*Colletotrichum gloeosporioides*
667829377	*Coniosporium apollinis*
152937537	*Cordyceps brongniartii*

109628446	*Cordyceps cardinalis*
573976171	*Cordyceps militaris*
671145855	*Cyphellophora europaea*
94411394	*Diaporthe eres*
629675377	*Eutypa lata*
684162144	*Exophiala dermatitidis*
758195067	*Fusarium graminearum*
685872678	*Fusarium pseudograminearum*
27960766	*Glomerella cingulata*
760173893	*Graphium penicillioides*
724472930	*Grosmannia clavigera*
27960782	*Hypocrea lutea*
396461041	*Leptosphaeria maculans*
110810461	*Melanconis stilbostoma*
629684016	*Metarhizium acridum*
761845151	*Metarhizium robertsii*
94411527	*Microascus trigonosporus*
398399249	*Mycosphaerella graminicola*
302921063	*Nectria haematococca*
615398955	*Neofusicoccum parvum*
119496508	*Neosartorya fischeri*
758979797	*Neurospora crassa*
698996823	*Neurospora tetrasperma*
255935410	*Penicillium chrysogenum*
212527771	*Penicillium marneffei*
630011474	*Pestalotiopsis fici*
169616974	*Phaeosphaeria nodorum*
171684990	*Podospora anserina*
631373337	*Pseudocercospora fijiensis*
27960780	*Pseudonectria rousseliana*
330917620	*Pyrenophora teres*
189188583	*Pyrenophora tritici-repentis*
636581076	*Setosphaeria turcica*
336264741	*Sordaria macrospora*
242784620	*Talaromyces stipitatus*
367042017	*Thielavia terrestris*
631242603	*Togninia minima*
589104066	*Trichoderma reesei*
21540003	*Trichophyton rubrum*

114150196	*Valsa ambiens*
697073754	*Verticillium dahliae*
comp31*	*Corollospora maritima*

*This sequence corresponds to the transcriptome reported in this work
