# Supplementary figures and images for: Comparative Transcriptome Analysis of the Cosmopolitan Marine Fungus Corollospora maritima Under Two Physiological Conditions
Source: G3 (Bethesda). 2015 Jun 26;5(9):1805–14. doi: 10.1534/g3.115.019620 (PMC4555217; doi:10.1534/g3.115.019620)

# GO Biological process

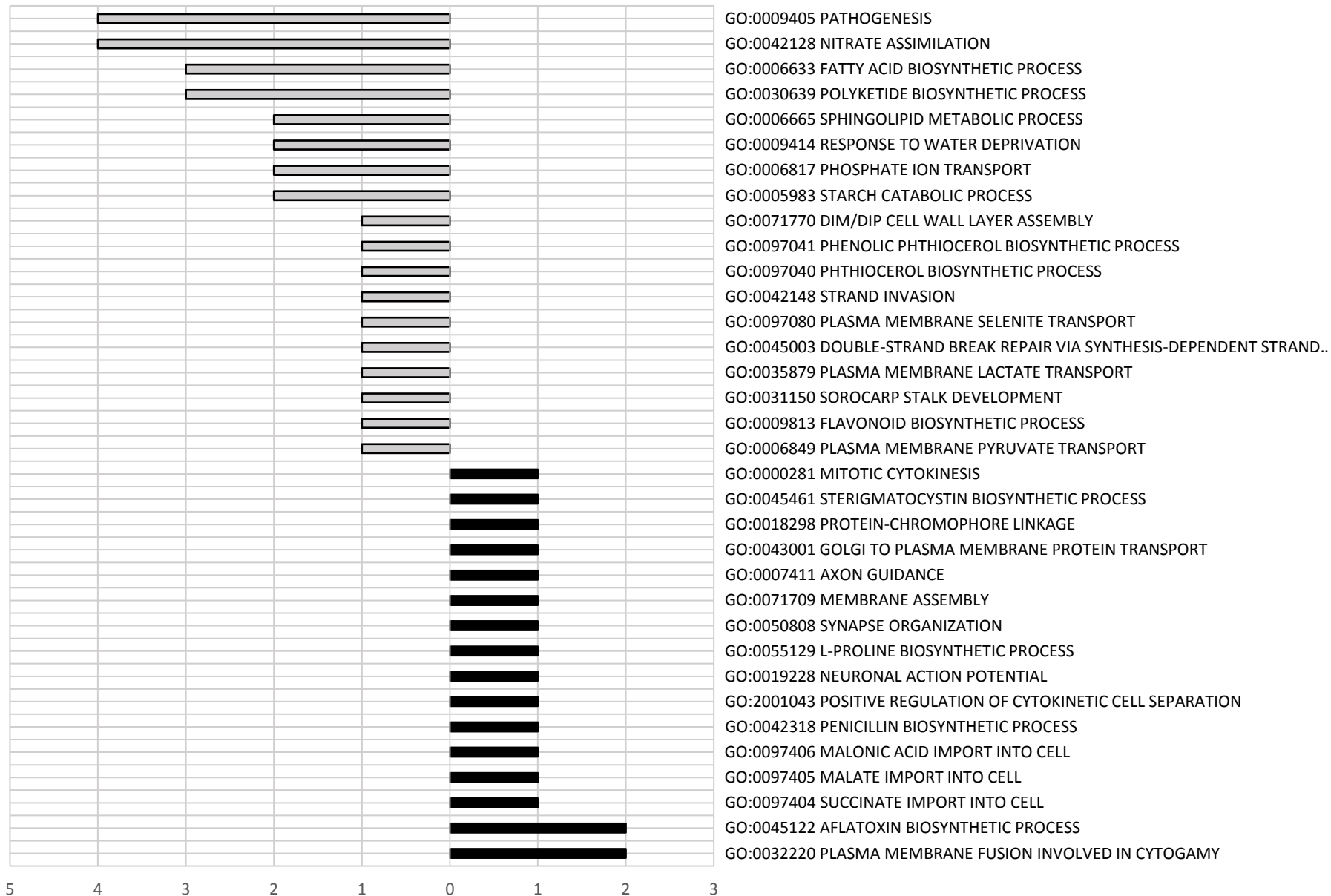

Supplement: Supporting Information [file supp_g3.115.019620_FileS3.pdf]

# GO Cellular component

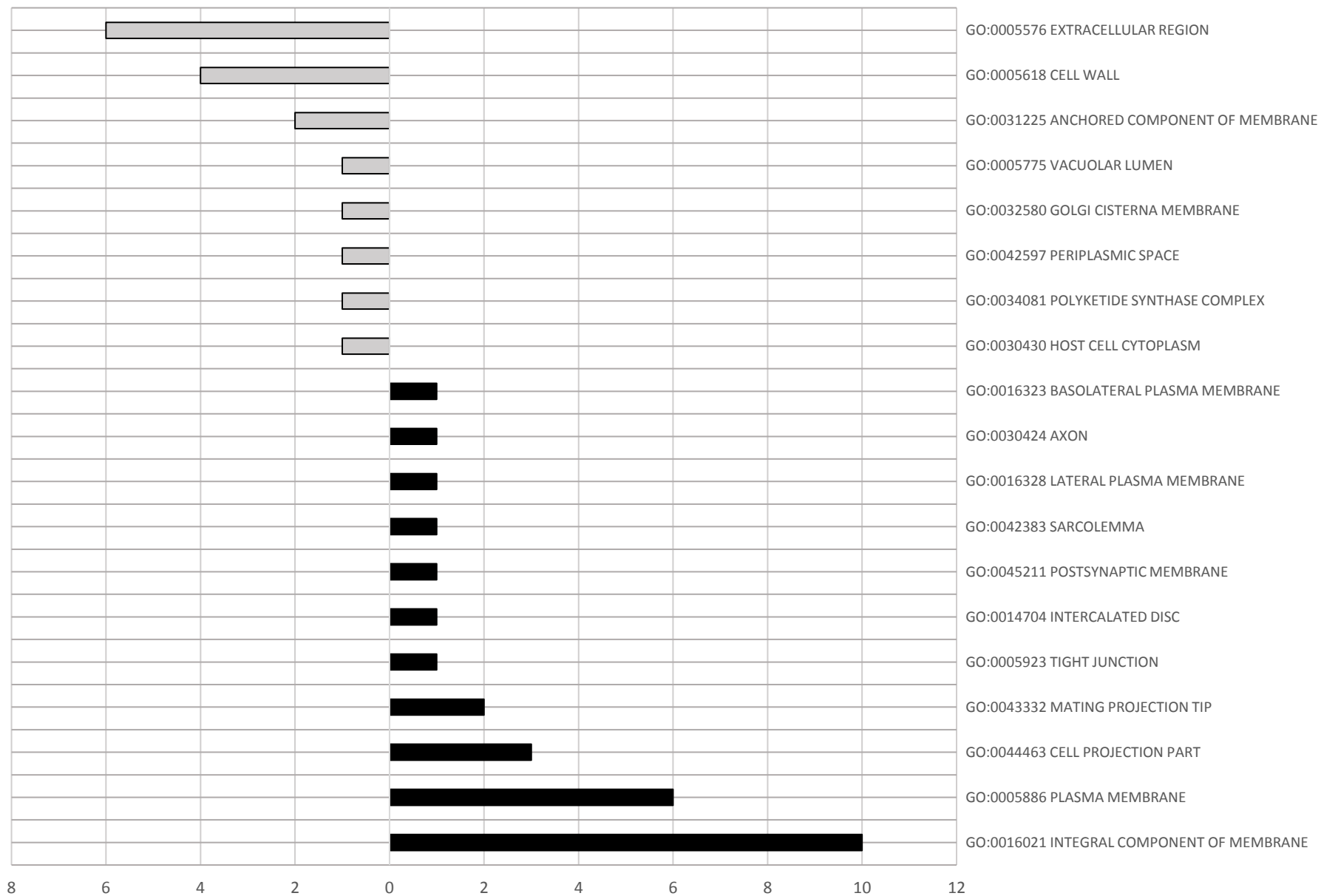

Supplement: Supporting Information [file supp_g3.115.019620_FileS4.pdf]

# GO Molecular function

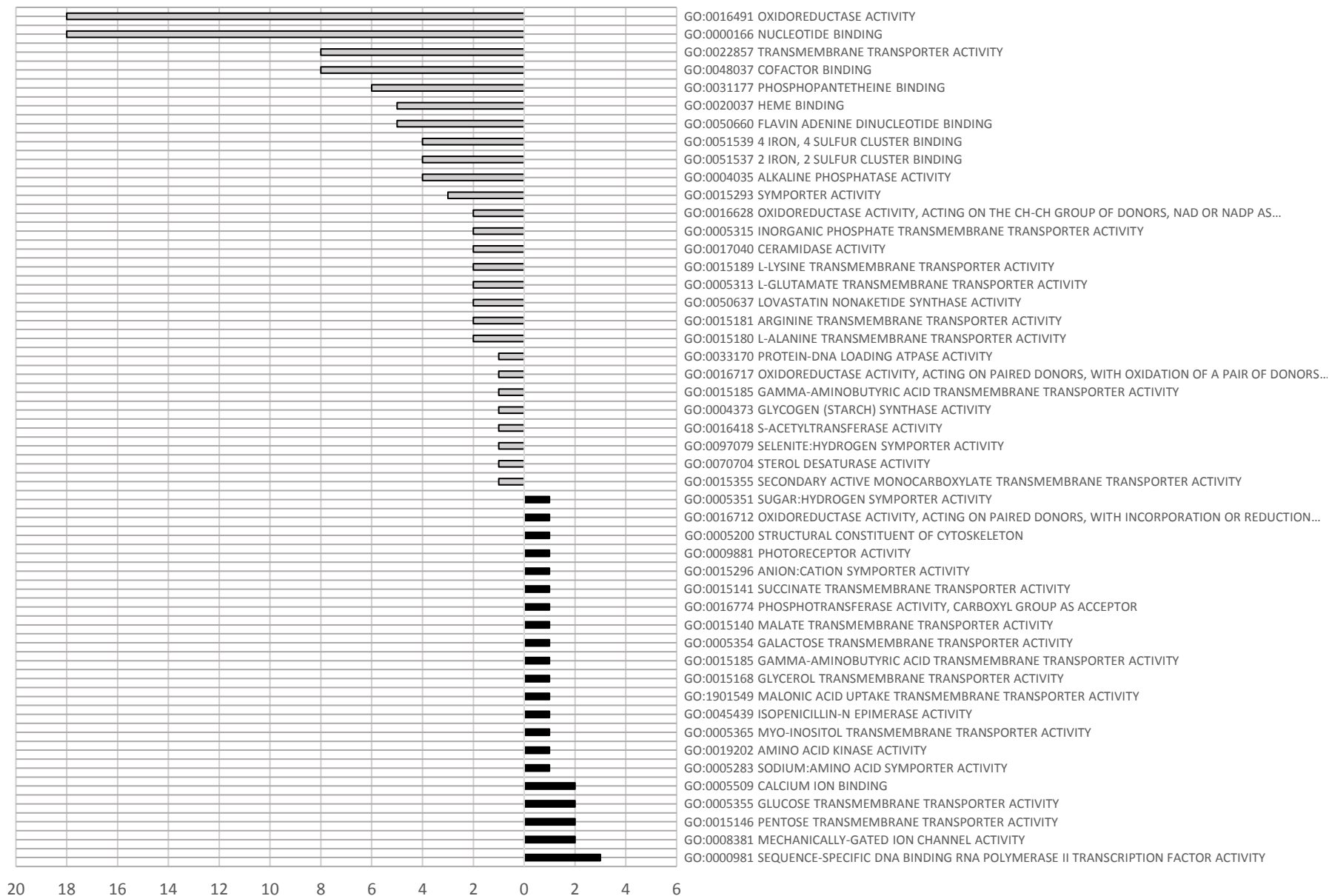

Supplement: Supporting Information [file supp_g3.115.019620_FileS5.pdf]
