# Supplementary material for: Comparative Transcriptome Analysis of the Cosmopolitan Marine Fungus Corollospora maritima Under Two Physiological Conditions
Source: G3 (Bethesda). 2015 Jun 26;5(9):1805–14. doi: 10.1534/g3.115.019620 (PMC4555217; doi:10.1534/g3.115.019620)
Supplement: Supporting Information [file supp_g3.115.019620_FileS2.zip › FileS2.html]

Javascript must be enabled to view this page.

members
magnitude

listaKrona

6152

445

445

445

445

104

104

1

1

24

23

1

10

8

1

1

69

69

1

1

1

1

1

1

1

1

25

7

1

1

2

2

1

1

1

1

1

1

1

1

10

10

1

2

1

1

1

1

2

1

1

1

1

4

1

1

1

1

1

1

1

1

3

3

2

1

314

49

1

1

48

15

33

4

2

2

1

1

1

1

1

1

1

4

4

4

73

73

3

70

2

2

2

1

1

1

86

86

86

8

8

8

42

42

6

2

28

3

3

14

14

14

1

1

1

10

5

5

5

5

8

8

8

11

11

11

5399

300

2

2

2

2

2

2

298

212

7

7

4

4

2

2

1

1

1

1

1

1

196

196

196

196

2

2

2

2

5

1

1

1

2

2

2

2

2

2

1

1

1

1

86

2

1

1

1

1

1

1

84

84

1

1

1

1

10

10

1

1

69

1

68

2

2

599

2

2

2

2

2

2

1

1

1

1

1

1

56

1

1

1

1

1

54

53

48

48

42

3

2

1

1

1

1

4

1

1

1

1

2

2

1

1

1

1

1

1

1

1

1

35

35

35

34

34

6

28

1

1

1

505

53

1

1

1

1

1

1

1

1

1

1

1

1

1

1

1

1

2

1

1

1

1

1

1

47

47

45

45

1

1

1

1

427

1

1

1

1

187

187

187

187

5

3

3

3

2

2

2

51

51

51

51

5

5

5

5

178

173

173

172

1

1

1

1

3

3

3

1

1

1

25

24

24

24

24

1

1

1

1

108

108

108

108

108

108

108

25

1

1

1

1

1

1

18

1

1

1

1

1

17

11

11

4

3

1

7

7

3

3

3

3

3

3

3

3

6

6

6

6

6

5

1

4367

30

4

4

4

4

4

20

5

5

5

5

2

2

2

2

13

6

1

1

5

5

4

1

1

3

1

2

1

1

1

2

2

2

6

6

6

6

6

4328

36

3

1

1

1

2

1

1

1

1

33

7

7

7

26

4

1

3

20

20

2

2

6

6

6

4

4

1

1

1

1

864

3

3

1

1

2

2

1

1

1

1

210

22

18

7

1

9

1

4

3

1

184

165

149

7

8

1

19

19

1

1

1

3

1

1

1

1

1

1

650

88

2

2

61

61

20

3

9

7

1

5

5

52

52

52

1

1

1

509

503

502

1

6

4

2

1789

1789

1789

1789

1789

1217

1217

1057

1

1

4

3

1

1

1

22

22

4

1

3

1

1

996

1

1

9

985

28

25

2

1

49

48

48

1

1

3

3

3

78

71

5

1

3

4

6

1

50

1

7

1

6

8

7

3

1

1

22

4

4

14

14

1

1

3

3

4

4

4

4

4

15

14

14

14

14

1

1

1

1

397

28

5

5

1

4

1

1

1

7

7

6

1

15

10

7

2

1

5

2

1

2

369

369

94

2

77

15

97

22

1

12

7

8

24

23

9

3

1

1

2

1

1

169

169

8

8

6

1

1

1

1

1

1

1

1

1

3

3

1

1

1

1

1

1

1

1

1

1

1

1

1

1

1

1

1

12

12

12

3

3

3

3

3

4

1

1

1

1

3

3

3

3

1

1

1

1

1

2

2

2

2

1

1

2

2

1

1

1

1

1

1

296

296

3

3

3

2

1

1

1

1

1

1

1

3

3

3

3

3

3

35

35

35

35

6

1

2

3

2

2

14

2

12

1

1

1

1

2

2

1

1

2

2

2

2

1

1

3

3

3

2

2

1

1

1

1

1

1

1

1

1

1

1

10

10

6

6

6

6

4

4

4

4

2

2

2

2

2

2

9

9

9

9

7

6

1

1

1

1

1

84

73

69

3

3

2

1

8

2

1

1

6

1

1

1

3

58

57

50

3

1

2

1

1

1

4

1

1

1

3

3

1

1

1

11

10

1

1

1

5

5

2

1

1

1

1

1

1

1

1

1

1

1

1

1

1

1

1

1

1

1

6

1

1

1

1

1

5

1

1

1

1

4

4

4

3

1

5

5

5

5

3

1

1

1

1

1

1

1

30

30

28

1

1

1

1

1

1

2

2

2

3

1

1

1

1

1

1

6

6

6

1

1

1

1

1

1

13

13

6

1

6

2

2

2

1

1

2

2

2

2

2

2

104

77

4

4

3

2

1

1

1

5

5

4

1

1

2

1

1

59

59

20

1

4

1

2

9

1

2

1

1

21

1

17

3

2

2

13

13

2

1

1

6

6

1

1

1

1

4

4

2

2

1

1

1

1

1

1

1

1

18

1

1

1

1

4

3

3

3

1

1

1

1

1

1

1

1

1

1

1

11

1

1

1

8

7

1

2

4

1

1

1

1

1

1

1

1

2

2

2

2

1

1

7

7

1

1

1

3

1

1

2

2

3

3

3
